# Osteoporotic quality of life, self-efficacy, and fracture protection behaviors in postmenopausal women

**DOI:** 10.1007/s11657-024-01377-4

**Published:** 2024-04-02

**Authors:** Gamze Ünver, Aysun Özlü, Ahmet Erdoğan, Muhammed Fatih Özdemir, Sema Üstündağ

**Affiliations:** 1 Faculty of Health Sciences, Department of Internal Medicine Nursing, Kutahya Health Sciences University, Kutahya, Turkey; 2 Faculty of Medicine, Department of Physical Medicine and Rehabilitation, Kutahya Health Sciences University, Kutahya, Turkey

**Keywords:** Osteoporosis, Menopause, Women

## Abstract

***Summary*:**

It is important for postmenopausal women to acquire bone health protective behaviors to protect them from fractures. For this reason, it is necessary to evaluate bone health during menopause and to inform women.

**Purpose:**

This study was conducted to examine osteoporotic fracture protection behaviors, quality of life, and self-efficacy in postmenopausal women.

**Methods:**

In the study, the data were evaluated with the socio-demographic data form, Osteoporotic Fracture Protection Scale, Osteoporosis Self-Efficacy-Efficacy Scale, European Osteoporosis Foundation Quality of Life Questionnaire-41, which includes introductory information on socio-demographic characteristics.

**Results:**

It was determined that the postmenopausal women included in our study were between the ages of 45–92; more than half of them had chronic diseases; their average BMI was 29; and their DEXA score was − 3.00 ± 0.41. Among the people included in our study, those with a history of fractures had lower self-efficacy scores. It was determined that the fracture prevention scale scores of the participants were above the average, and the average of the osteoporosis-related quality of life score was high. In addition, it was determined that there was a strong positive correlation between self-efficacy and fracture prevention scale.

**Conclusion:**

It is important to determine behaviors to prevent osteoporotic fractures in postmenopausal women, to raise the necessary awareness and to inform patients about the precautions to be taken. It is thought that it will increase patients’ quality of life by increasing their disease-related self-efficacy. Therefore, there is a need for research on providing education to op patients and examining the results.

## Introduction

Osteoporosis is defined as a systemic skeletal disease characterized by low bone mass and disruption of the microarchitecture of bone tissue, resulting in increased bone fragility and fracture risk. With accelerated bone loss after menopause, bone mass decreases with age, and the risk of osteoporotic fractures increases [1]. Life expectancy in the world has been increasing in recent years [2, 3]. The population of our country is not a young population with high fertility and mortality rates; it has low fertility and mortality rates and is getting older [4, 5]. According to the 2014 data of the Turkish Statistical Institute (TUIK), the rate of the elderly population in our country is 8%. It is estimated that it will increase to 10.2% in 2023, 20.8% in 2050, and 27.7% in 2075 [5, 6]. The rates of chronic diseases also increased with advancing age. In multicenter studies conducted throughout Turkey, chronic diseases frequently seen in the elderly are, respectively, hypertension 30.7%, osteoarthritis 13.7%, chronic heart failure 13.7%, diabetes mellitus 10.2%, coronary artery disease 9.8%, and osteoporosis 8.2% [7]. Especially in postmenopausal women, osteoporotic fractures occur due to decreased bone density. Major osteoporotic fractures include fractures of the vertebrae, hip, distal forearm, and proximal humerus osteoporotic fractures. It causes a decrease in the quality of life, being bedridden for a long time, an increase in health expenditures, surgical operations or death as a result [8].

In the coming years, it is estimated that osteoporotic fractures will significantly affect postmenopausal women in all countries of the world. The risk of osteoporotic fractures nearly doubles every 5–7 years due to osteoporosis. It is thought that the prevention and treatment of osteoporotic fractures will become a serious public health problem in the coming years. Strategies to protect from fracture are constantly evolving on how to manage osteoporotic fractures when they occur [9, 10].

Osteoporosis increases the risk of fractures associated with increased mortality and lower quality of life. At age 50, the lifetime risk of osteoporotic fractures is close to 50% for women and greater than 20% for men. The clinical significance of osteoporosis is determined by subsequent fractures leading to deterioration in quality of life [8]. It is important to determine the behaviors to prevent osteoporotic fractures in postmenopausal women, to raise the necessary awareness, and to inform the patients about the precautions to be taken. This study was carried out to examine the osteoporotic fracture prevention behaviors and quality of life self-efficacy status in postmenopausal women.

## Material and method

This cross-sectional study was conducted between June 2022 and December 2022. A total of 100 postmenopausal women who applied to the Kutahya Health of Science University physical therapy and rehabilitation outpatient clinic in Turkey due to musculoskeletal pain were included in the study. The patients included in the study were diagnosed at least 1 year ago.

The 100 postmenopausal women screened for osteoporosis with DEXA were included in the study. Participants with a DEXA score of lumbar and/or femur T score of − 2.5 and below and diagnosed with osteoporosis were included in the study. DEXA evaluation, which is routinely done in clinical practice, was performed on all those participating in the study.

A diagnosis of osteoporosis in patients whose bone mineral density (BMD) was measured by dual-energy X-ray absorptiometry (DEXA) (GE LUNAR DPX-NT) was made. It will be determined by evaluating the T score based on the World Health Organization criteria. In the study, BMD values with a bone mineral density T score of the lumbar region and femoral neck T score below − 2.5 SD compared to the average of same-sex adults in at least one region were accepted as “osteoporosis.”

### Data collection tools

In the study, the data were evaluated with the socio-demographic data form, Osteoporotic Fracture Protection Scale, Osteoporosis Self-Efficacy-Efficacy Scale, European Osteoporosis Foundation Quality of Life Questionnaire-41, which includes introductory information on socio-demographic characteristics.

### Socio-demographic data form

Participants included socio-demographic characteristics and features that may affect fracture development (activity in daily work, fall history and time, fall-related fracture status and region, activity status, experience of falling and fracture, orthopedic surgery, use of assistive orthopedic tools, and diagnosis of osteoporosis and osteoporosis). Time was evaluated with a questionnaire about the DEXA score.

#### OFPS

An Osteoporotic Fracture Prevention Scale (OFPS), developed in 2015 based on the planned behavior model, was used to evaluate the intention and behavior towards prevention of osteoporotic fracture. The scale includes 22 five-point Likert propositions. OFPS consists of 6 sub-headings: belief, attitude and behavior control, diagnosis and treatment behavior, disability perception, fall prevention intention, and diagnosis and treatment intention. It contains recommendations on bone mineral density measurement for diagnosis and follow-up, drug treatment, following calcium-rich diet and sunbathing recommendations, daily physical activity and/or regular physical exercise, and making home arrangements to prevent falls. The internal consistency of the scale is Cronbach’s a coefficient of 0.95. The lowest score that can be obtained from the scale is 22, and the highest score is 110. A high score indicates the high level of protection from osteoporotic fracture. The scale was found to be highly valid and reliable in Turkish population. It can be a part of the public health model aimed at preventing osteoporotic fractures [10].

#### QUALEFFO-41

European Osteoporosis Foundation Quality of Life Questionnaire-41 (Quality of Life Questionnaire of the European Foundation for Osteoporosis-41, QUALEFFO-41) is a self-assessment scale that is widely used in the evaluation of OP-specific quality of life; it is reproducible and can clearly reveal the differences between the patients and the control group. This scale consists of 41 questions in the subgroups of pain (5 questions), physical function (17 questions), social function (7 questions), general health assessment (3 questions), and mental function (9 questions). While 0 points for total score and subgroup scores indicate the best health status, higher scores mean poor quality of life. Turkish validity and reliability were evaluated by Koçyiğit et al. [11].

The answers to the questions in the QUALEFFO-41 scale are scored from 1 (healthy) to 5 (unhealthy), respectively; between 1 and 3 for questions 23, 24, 25, and 26; for questions 27, 28, and 29, between 1 and 4. The remaining questions were scored between 1 and 5. For questions 24, 26, and 29, no points were given for the options “this question does not apply to me” or “I do not go to the cinema or the theater.” While scoring the 33rd, 34th, 35th, 37th, 39th, and 40th questions, the order of the options is reversed so that the order is from the best health (1 point) to the worst health (5 points), as in the other questions. The section score and the total score are calculated by transferring the scores to a formula made out of 100. With this formula, each subscale and total QUALEFFO-41 scores are calculated. For each subgroup and total result in the scale, 0 points indicate the best health status, while 100 points indicate the worst health status. The formula used is as follows [12, 13].

### Osteoporosis Self-Efficacy Scale

It was developed by Gendler, Horan and Kim, and its Turkish validity and reliability were made by Kılıç. Marking on a 12-item visual-like scale is done on a scale that includes numbers from 0 to “I have no confidence at all” to 10 “I have a lot of confidence.” A score between 0 and 100 is taken as basis for each item. The total score of the scale is 0 at the lowest and 1200 at the highest. Cronbach alpha reliability coefficient ranges between 0.96 and 0.98.

#### Osteoporosis Exercise Self-Efficacy Scale

It determines the degree of perceived confidence in performing exercises and activities for the Prevention of Osteoporosis Scale. It includes items 1, 2, 3, 4, 5, and 6.

#### Osteoporosis Calcium Self-Efficacy Scale

The scale determines the degree of perceived confidence in carrying out activities related to calcium intake for the Prevention of Osteoporosis Scale. It contains items 7, 8, 9, 10, 11, and 12 [12, 14].

### Inclusion criteria

- Postmenopausal women
- Able to communicate in Turkish
- It was planned to include individuals who were literate and who agreed to participate in the study among patients without a psychiatric illness.

### Exclusion criteria

- Those with severe hearing loss or visual impairment, who have undergone major surgery in the last 2 months, with extremity amputation, who are scheduled for surgery soon, and with acute trauma
- Patients who have surgically entered menopause
- Those who are treated for malignancy
- Terminal stage cancer patients
- Those who have literacy and comprehension problems
- Those with communication problems and those who did not agree to participate in the research were not included in the research.

### Statistical analysis

Frequency tables and descriptive statistics were used to interpret the findings for statistical analysis (Table 1). The “Student’s *t* test” *t*-table value was used to compare the scale score averages of the independent variables with normal distribution. Pearson correlation “*r*” coefficient was used for normally distributed averages in the comparison of the relationship between the scale point averages according to the research question.

**Table 1:** Demographic characteristics of the patients

	*N* = 100
	*n* (%)
Age ($\overline{{\text{X}}}\pm \mathrm{S }.{\text{S}}$.) [min–max]	65.3 ± 9.79 [45–92]
BMI ($\overline{{\text{X}}}\pm \mathrm{S }.{\text{S}}.$) [min–max]	29.10 ± 5.03 [17.58–39.11]
DEXA ($\overline{{\text{X}}}\pm \mathrm{S }.{\text{S}}.$) [min–max]	− 3.00 ± 0.41 [− 3.90 to − 2.50]
Marital status	
Married	82 (82.2)
Single	18 (18.0)
Educational status	
Primary education	37 (37.0)
Secondary education	56 (56.0)
University Degree	7 (7.0)
Family	
Lives alone	25 (25.0)
Nuclear family	51 (51.0)
Extended family	24 (24.0)
Economical situation	
Income less than expenses	6 (6.0)
Income equals expense	70 (70.0)
Income more than expenses	24 (24.0)
Working status	
Working	25 (25.0)
Not working	75 (75.0)
Number of children	
1	6 (6.0)
2	49 (49.0)
3	39 (39.0)
4	6 (6.0)
Activity in daily work	
Yes	77 (77.0)
No	23 (23.0)

## Results

It was determined that the participants were 65.3 ± 9.79 years old on average; their average BMI was 29.10 ± 5.03; and their DEXA score was − 3.00 ± 0.41. It was determined that the majority of the participants were married; more than half of them were secondary school graduates; and 77% of them were engaged in activities in their daily work.

It was determined that the mean disease duration of the participants was 9.01 ± 5.41 years; 62% had a history of falling; nearly half had a history of fracture; and 88% had a chronic disease. It was determined that the majority of the patients did not smoke or use alcohol (Table 2).

**Table 2:** Disease status characteristics of the participants

	*N* = 100
	*n* (%)
Osteoporosis disease duration (years) (X ® ± S.S.) [min–max]	9.01 ± 5.41 [1–20]
Chronic disease (CH)	
There is	88 (88.0)
None	12 (12.0)
Smoking	
There is	19 (19.0)
None	81 (81.0)
Alcohol	
There is	6 (6.0)
None	94 (94.0)
Surgical history	
There is	19 (19.0)
None	81 (81.0)
Assistive device usage	
Yes	6 (6.0)
No	94 (94.0)
Drugs used by those with CH	
Anti-hypertensive	63 (63.0)
Anti-diabetic	37 (37.0)

It was determined that the Osteoporosis Self-Efficacy Scale score of the participants was close to the average (min 0–max 120) (Table 3). It was determined that the participants’ Osteoporotic Fracture Prevention Behavior Scale scores were above the average (min 22–max 110). It was determined that the European Osteoporosis Quality of Life Scale score average of the participants was quite high (min 0–max 100).

**Table 3:** Osteoporosis Self-Efficacy, Fracture Prevention Behaviors and Quality of Life Scale Scores

	($$\overline{{\text{X}}}\pm \mathrm{S }.{\text{S}}.$$) [min–max]
Osteoporosis Self-Efficacy Scale (OSES)	
Osteoporosis Self-Efficacy total	Osteoporosis Self-Efficacy-Efficacy total
Osteoporosis Exercise Summary — sub-clause	Osteoporosis Exercise Summary — sub-clause
Osteoporosis Calcium Summary — sub-article	Osteoporosis Calcium Summary — sub-article
Osteoporotic Fracture Prevention Behaviors	
Osteoporotic Fracture Prevention Scale (OFPS)	Osteoporotic Fracture Prevention Scale (OFPS)
Evaluation of Quality of Life	
European Osteoporosis Quality of Life Questionnaire-41 (QUALEFFO-41)	European Osteoporosis Quality of Life Questionnaire-41 (QUALEFFO-41)
QUALEFFO-41 — Total	QUALEFFO-41 — Total
Quality of Life — Pain	Quality of Life — Pain
Quality of Life — Physical function	Quality of Life — Physical function
Quality of Life — Social function	Quality of Life — Social function
Quality of Life — General health	Quality of Life — General health
Quality of Life — Mental function	Quality of Life — Mental function

When the relationship between the participants’ Fracture Prevention Behaviors, Quality of Life and Self-Efficacy Scales was examined, it was determined that there was a strong positive relationship between OSES and OFPS and a strong negative relationship between OSES and QUALEFFO (Table 4). It was determined that there was a positive relationship between the participants’ DEXA scores and OSES and a negative relationship between QUALEFFO.

**Table 4:** Correlation between participants’ Fracture Prevention Behaviors, Quality of Life, and Self-Efficacy Scales

	OSES (*r*)	OFPS (*r*)	QUALEFFO (*r*)
OSES	1	0.589**	− 0.418**
OFPS	0.589**	1	− 0.124
QUALEFFO	− 0.418**	− 0.124	1
DEXA	0.255*	− 0.060	− 0.467**

Pearson correlation * *p* < 0.05 ** *p* < 0.01

It was determined that the participants who were active in their daily work had higher OSES scores and lower QUALEFFO scores (Table 5). Participants with a history of falling had lower OSES and OFPS scores but higher QUALEFFO. Participants with a history of fractures had lower OSES scores. In addition, participants with a history of fracture were found to have higher QUALEFFO. Participants using assistive devices had lower OSES and OFPS scores but higher QUALEFFO scores.

**Table 5:** Fracture prevention behaviors, quality of life, and self-efficacy-efficacy status of the participants in terms of some variables

		OSES	OFPS	QUALEFFO
		($\overline{{\text{X}}}\pm \mathrm{S }.{\text{S}}.$)	($\overline{{\text{X}}}\pm \mathrm{S }.{\text{S}}.$)	($\overline{{\text{X}}}\pm \mathrm{S }.{\text{S}}.$)
Activity status in daily work Yes No	Activity status in daily work Yes	66.03 ± 24.04	85.29 ± 13.98	72.35 ± 17.81
	No	48.08 ± 27.64	80.73 ± 12.95	88.27 ± 28.77
Test statistic*		Test statistic*	1.395	− 2.459
*p*		*p*	0.166	**0.021**
Has a history of falling None	Has a history of falling	51.83 ± 22.79	79.90 ± 13.56	83.16 ± 23.60
	None	78.34 ± 22.17	91.34 ± 11.16	62.94 ± 7.58
Test statistic*		Test statistic*	− 4.367	6.188
*p*		*p*	0.000	0.000
Has a history of fracture None	Has a history of fracture	50.00 ± 21.40	81.43 ± 10.35	86.09 ± 25.77
	None	71.26 ± 25.43	86.46 ± 15.77	77.46 ± 12.54
Test statistic*		Test statistic*	− 1.918	4.376
*p*		*p*	0.071	**0.000**
Assistive device usage status Yes None	Assistive device usage status Yes	32.00 ± 13.89	79.00 ± 3.09	81.00 ± 0.00
	None	63.81 ± 25.35	84.58 ± 14.17	10.22 ± 16.68
Test statistic*		Test statistic*	− 2.888	33.956
*p*		*p*	**0.008**	**0.000**

* Student’s *t*-test

## Discussion

It was determined that the postmenopausal women included in our study were between the ages of 45–92; more than half of them had chronic diseases; their average BMI was 29; and their DEXA score was − 3.00 ± 0.41. Low bone density is known to be associated with a higher fracture rate, and many studies show an association between early menopause, oophorectomy, and an increase in osteoporotic fractures [15].

Among the participants included in our study, those with a history of fracture had lower OSES scores. In Akpınar’s study, 46% of women experienced a fall. Fractures developed in approximately one third of those who fell [16]. Çıtıl et al. reported that 50% of women fell within the last month; 23% fell more than once; and 9% suffered fractures as a result of falling [17]. It is thought that the level of self-efficacy is affected, and the quality of life is affected due to the high rates of fractures and negative health outcomes.

In a study conducted with 419 women between the ages of 19 and 60 with different education levels, it was reported that the osteoporosis self-efficacy level was low [18]. Arslan et al. found that the OSES scores of the participants were low in their study, which included 236 individuals over the age of 35, 53.4% female and 34.7% literate, who applied to the bone densitometry unit of a hospital [19].

It was determined that the OFPS scores of the participants were above the average. The Centers for Disease Control determined people who needed screening. Screening is recommended for Caucasian women aged 65 years or above without any additional risk factors, while it is recommended for younger women who are at risk for OP and fractures [20]. The OSES scores of the participants included in our study were found to be close to the average. It was determined that the average QUALEFFO score of the participants was high. While 0 points for total score and subgroup scores indicate the best health status, higher scores mean poor quality of life. It was determined that there was a strong positive relationship between OSES and OFPS of the participants and a strong negative relationship between OSES and QUALEFFO. In the study of Ahn and Oh, which included elderly women, it was determined that there was a positive relationship between osteoporosis exercise self-efficacy and diet self-efficacy and protective behaviors from osteoporosis [21]. Wu and Sheng conducted a study with elderly individuals and determined that there is a positive relationship between “self-efficacy” and “healthy lifestyle behaviors” for health practices [22]. Guntzviller et al. reported that exercise self-efficacy is associated with exercise behavior in low-income adults [23]. It is known that physically inactive individuals have a higher incidence of osteoporosis and related complications [24]. In the study of Arslan et al., it is shown that the higher the education level, the higher the osteoporosis self-efficacy-efficacy level [19]. When the results are examined, it is thought that people with high osteoporosis self-efficacy can develop fracture prevention behaviors, and their quality of life is affected by this situation. In addition, it is known that the level of self-efficacy is affected by education level and socioeconomic status.

## Conclusion

It is important to determine behaviors to prevent osteoporotic fractures in postmenopausal women, to raise the necessary awareness and to inform patients about the precautions to be taken. It is thought that it will increase patients’ quality of life by increasing their disease-related self-efficacy. Therefore, there is a need for research on providing education to op patients and examining the results.
